# The Association between Cerebral White Matter Lesions and Plasma Omega-3 to Omega-6 Polyunsaturated Fatty Acids Ratio to Cognitive Impairment Development 

**DOI:** 10.1155/2015/153437

**Published:** 2015-10-25

**Authors:** Michihiro Suwa, Shigeru Yamaguchi, Tsuyoshi Komori, Sachiko Kajimoto, Masaya Kino

**Affiliations:** ^1^Department of Cardiology, Hokusetsu General Hospital, Takatsuki, Osaka 569-8585, Japan; ^2^Department of Radiology, Hokusetsu General Hospital, Takatsuki, Osaka 569-8585, Japan; ^3^Department of Rehabilitation, Aijinkai Rehabilitation Hospital, Takatsuki, Osaka, Japan

## Abstract

*Objective*. Cerebral white matter hyperintensity (WMH) with magnetic resonance imaging (MRI) has a potential for predicting cognitive impairment. Serum polyunsaturated fatty acid (PUFA) levels are important for evaluating the extent of atherosclerosis. We investigated whether abnormal PUFA levels affected WMH grading and cognitive function in patients without significant cognitive impairment. *Methods*. Atherosclerotic risk factors, the internal carotid artery (ICA) plaque, and serum ratios of eicosapentaenoic to arachidonic acids (EPA/AA) and docosahexaenoic to arachidonic acids (DHA/AA) were assessed in 286 patients. The relationship among these risk factors, WMH, and cognitive function was evaluated using WMH grading and the Mini-Mental State Examination (MMSE). *Results*. The development of WMH was associated with aging, hypertension, ICA plaques, and a low serum EPA/AA ratio (<0.38, obtained as the median value) but was not related to dyslipidemia, diabetes, smoking, and a low serum DHA/AA ratio (<0.84, obtained as the median value). In addition, the MMSE score deteriorated slightly with the progression of WMH (29.7 ± 1.0 compared to 28.4 ± 2.1, *P* < 0.0001). *Conclusions*. The progression of WMH was associated with a low serum EPA/AA ratio and accompanied minimal deterioration in cognitive function. Sufficient omega-3 PUFA intake may be effective in preventing the development of cognitive impairment.

## 1. Introduction

Cerebral white matter hyperintensities (WMHs) on magnetic resonance imaging (MRI) increase in the prevalence and the degree with age [1]. However, WMHs are also associated with cerebral small vessel disease affecting the same region of the brain [2]. Thus, the progression of small vessel disease is common and more extensive in patients with cardiovascular or cerebrovascular diseases and atherosclerotic risk factors [3]. Currently, several observational studies have reported that the progression of WMH and lacunes is a better predictor for the development of cognitive impairment [3–6].

There is a lower incidence of subclinical ischemic stroke in elderly patients who consume high levels of fish in their diet, compared to that in those who do not [7, 8]. In addition, a low serum eicosapentaenoic acid (EPA) level and a low EPA to arachidonic acid (AA) ratio were associated with unstableness of coronary artery disease [9]. EPA supplementation was found to be effective for the secondary prevention of coronary artery disease and in reducing the risk of recurrent stroke [9, 10]. Therefore, polyunsaturated fatty acid (PUFA) balance might act as an important diagnostic parameter for evaluating the extent of arteriosclerosis associated with coronary artery and cerebrovascular diseases [9, 10]. Finally, some investigations reported that patients with Alzheimer's disease (AD) had low serum omega-3 (*ω*3) PUFA levels and that high PUFA intake may postpone cognitive decline [11–13]. Also, the influence of vascular disturbance to cognitive impairment may be lessened through the improvement of *ω*3 PUFA level.

Therefore, we investigated whether low *ω*3 PUFA levels in the blood affected the development of WMH and influenced cognitive function in middle aged or presenile persons who did not develop clinically cognitive impairment.

## 2. Methods

### 2.1. Study Design

This study was carried out from January 2010 to June 2013 on 286 outpatients (161 men and 125 women, age range, 55–84 years) who had at least one atherosclerotic risk factor and accepted to have the following various examinations. Among them, 146 patients were having regular visiting with various cardiovascular diseases and were undergoing stable treatment without heart failure. Further 140 patients were visiting for health checkups and had complete physical and blood examinations for screening.

Clinical data were obtained from all patients in the outpatient clinic. Patients underwent physical examination, blood pressure measurement, chest radiography, electrocardiography, brain MRI, duplex ultrasonography of the carotid arteries, and laboratory examination including the measurement of fasting plasma levels of fatty acids and screening test for cognitive impairment using the Mini-Mental State Examination (MMSE), with the written informed consent. Patients with myocardial infarction, cardiomyopathies, stroke, documented paroxysmal or persistent atrial fibrillation and cardiac surgery for coronary artery diseases, or valvular heart diseases were excluded by history taking and physical and various examinations.

### 2.2. Study Patients

This study was approved by the review committee in Hokusetsu General Hospital. A total of 291 patients provided written informed consent.

Among these 286 patients, 30 had coronary artery disease, 197 exhibited hypertension with (*n* = 28) or without coronary artery disease, 192 had dyslipidemia with (*n* = 22) or without coronary artery disease, and 43 had diabetes with (*n* = 11) or without coronary artery disease.

The presence of hypertension was established if the patient had 1 of the following, systolic blood pressure >160 mmHg or diastolic pressure >90 mmHg, or if the patient used antihypertensive medications. The presence of dyslipidemia was established if the patient had a serum low-density lipoprotein level ≥160 mg/dL or if the patient received recent medication (mainly statin) for dyslipidemia. The presence of diabetes was established if the patient had a hemoglobin A1c value of ≥6.9% (NGSP) at entry or if the patient received medication for hyperglycemia. Any patient who was a former or current smoker was characterized as a smoker. The presence of coronary artery disease was established if coronary lesions had previously been identified by angiography or if the patients had undergone previous coronary intervention.

### 2.3. Serum Fatty Acids

Fasting serum levels of EPA, docosahexaenoic acid (DHA), and AA were measured using gas chromatography at an external laboratory (SRL Inc., Tokyo, Japan).

### 2.4. Brain MRI

MRI examinations, consisting of T1 (TR 4200 ms, TE 102 ms, 90° flip angle, and 3 excitations) and T2 (TR 2000 ms, TE 10 ms, TI 750 ms, 90° flip angle, and 2 excitations) weighted and FLAIR (TR 8000 ms, TE 150 ms, TI 2100 ms, 90° flip angle, and 1 excitations) images, were performed using an MRI with a 1.5-T magnet (GE SIGNA-LX, GE electronics, USA). Brain images were obtained from 13 transverse slices (5 mm slice thickness, 2.5 mm slice interval) on the base of the anterior commissure-posterior commissure line or orbitomeatal line.

Using the WMH grading scale (Supplementary Figure in Supplementary Material available online at http://dx.doi.org/10.1155/2015/153437), MRI FLAIR-defined deep and subcortical WMH (DSWMH: 5 grades from grade 0 to 4), and periventricular WMH (PVH: 5 grades from grade 0 to IV), released from the Japanese brain dock society (http://jbds.jp/guideline.html) outlined by reports of Shinohara et al. [14] and Fukuda and Kitani [15], all images were read and assessed by the same 2 investigators (1 cardiologist and 1 radiologist) who were blind to the study. The interobserver reliability of the 2 investigators using 50 MRI scans was 0.91 in DSWMH and 0.88 in PVH as Spearman's rank correlation.

### 2.5. Carotid Ultrasound

Carotid ultrasonography was performed by a brain surgeon, using a standard protocol and the same ultrasound equipment with a high-resolution linear-array transducer and color Doppler (9L probe, Vivid 7 Dimension, GE Health Care, Horten, Norway). The presence of a plaque was established when at least 1 protruded and localized high echo signal of more than 2 mm as intima-media thickness in the internal carotid artery (ICA) was observed.

### 2.6. Evaluation of Cognitive Function

The Japanese version of the MMSE was performed on a total of 216 patients on the next hospital visit after MRI examination. This cognitive examination was performed only in patients being older than age of 60 years and had agreed to do it. They were divided into 3 groups depending on their MRI results as follows: Group A: 74 patients with no or mild ischemic lesions of either hyperintensity (DSWMH grade 0 or 1 and PVH grade 0 or I), Group B: 84 patients with at least 1 moderate ischemic lesion of either hyperintensity (DSWMH grade 2 or PVH grade II), and Group C: 58 patients with at least 1 advanced MRI ischemic lesion of either hyperintensity (DSWMH grade 3 or 4 or PVH grade III or IV). MMSE scores were compared among these 3 Groups.

### 2.7. Statistical Analysis

Two-tailed statistical analysis was performed with a 5% level of significance. The Wilcoxon 2-sample test was used to compare continuous variables. The chi-square (*χ* ^2^) test was used to compare categorical variables. Ordered logistic regression analysis was used to assess the association of selected dichotomized (present or not, or above or less) variables including risk factors for atherosclerosis (hypertension, dyslipidemia, diabetes, smoking, ICA plaque, and the median value of serum EPA/AA and DHA/AA ratios) and the 5 grades of two types of WMH. MMSE were evaluated using regression analyses among the 3 groups (A to C) of hyperintensities mentioned above. These statistical analyses were performed using JMP pro, version 9.0.2 (SAS Institute, Cary, NC, USA). Data are expressed as the mean ± standard deviation (SD).

## 3. Results

**Figure 1:**
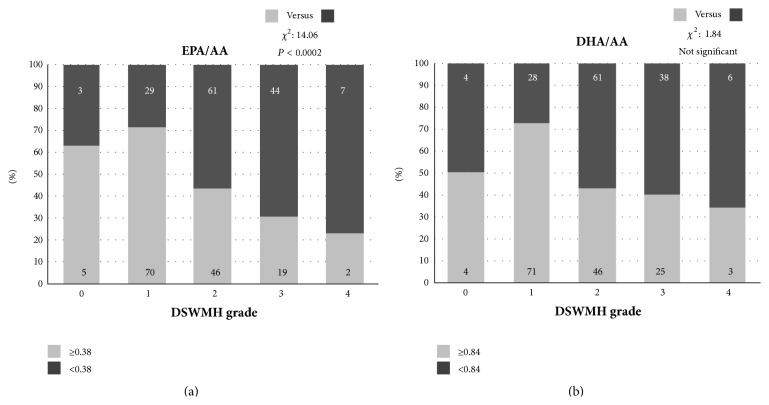
The distribution of patients with eicosapentaenoic acid to arachidonic acid (EPA/AA) ratios (a) and docosahexaenoic acid to arachidonic acid (DHA/AA) ratios (b) of more (white area in the column) and less (black area in the column) than each median value according to deep and subcortical white matter hyperintensity (DSWMH) grades 0–4. With the progression of DSWMH grade (figures in the column express patients numbers), the percentage of patients with EPA/AA ratios of less than 0.38 (black area on (a)) increased (chi-square: 43.64, *P* < 0.0001), and the percentage of those with DHA/AA ratios less than 0.84 (black area in (b)) also increased but did not reach statistical significance (chi-square: 1.02, no significance).

**Figure 2:**
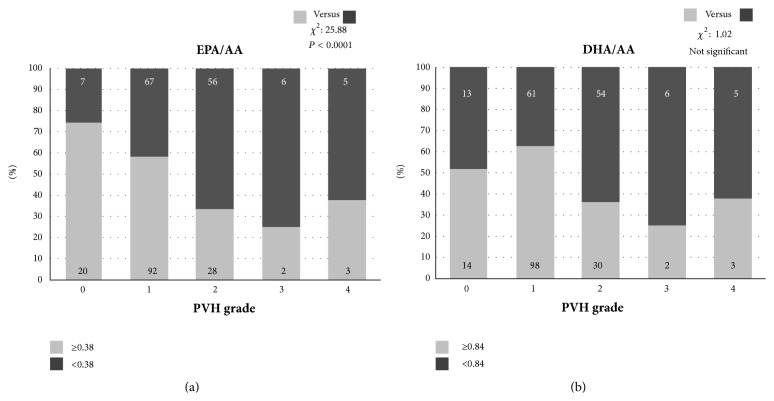
The distribution of patients with eicosapentaenoic acid to arachidonic acid (EPA/AA) ratios (a) and docosahexaenoic acid to arachidonic acid (DHA/AA) ratios (b) of more (white area in the column) and less (black area in the column) than each median value according to periventricular hyperintensity (PVH) grades 0–IV. With the progression of PVH grade (figures in the column express patients numbers), the percentage of patients with EPA/AA ratios of less than 0.38 (black area on (a)) increased (chi-square: 30.20, *P* < 0.0001), and the percentage of those with DHA/AA ratios less than 0.84 (black area in (b)) also increased but did not reach statistical significance (chi-square: 1.84, no significance).

The raw data of 3 FUFAs and their ratios according to WMH levels in the two WMH types (DSWMH and PVH) are expressed in Supplementary Table. In the current study, logistic regression analysis using the median values of *ω*3 to *ω*6 PUFA ratios (0.38 in serum EPA/AA ratio and 0.84 in DHA/AA ratio) demonstrated that the progression in DSWMH grade was associated with aging, female sex, the presence of hypertension and ICA plaque, and a low serum EPA/AA ratio (<0.38) but was not related to dyslipidemia, diabetes, smoking, and a low serum DHA/AA ratio (<0.84) (Figure 1, Table 1). In addition, the progression in PVH grade was also associated with aging, female sex, the presence of hypertension and ICA plaque, and a low serum EPA/AA ratio but was not related to the other 4 risk factors (Figure 2, Table 2).

MMSE scores, evaluated in only 216 patients, showed minimal but statistically significant reductions with the progression of MRI ischemic lesions (Table 3).

## 4. Discussion

The present study indicated that the progression of cerebral WMH was strongly related to low serum EPA levels as well as aging, female sex, hypertension, and the presence of ICA plaque, in presenile persons who did not develop clinically relevant cognitive impairment. Furthermore, cognitive function slightly deteriorated in these subjects with the progression of cerebral WMH.

With the development of medical technology, silent brain infarction, that is, WMH and lacunar viewed as cerebral small vessel disease, is being reported more frequently in older people [4]. The Rotterdam MRI scan study showed that newly developed silent brain infarcts were related to the presence of cardiovascular risk factors, such as hypertension, dyslipidemia, diabetes, carotid plaques, and smoking, in addition to the age factor [3]. Several reports revealed that the increasing severity of WMH and lacunar was related to a decline in cognitive function scoring in these patients [5, 6]. Disease risks for cardiovascular disease and development of cognitive impairment are the same [16–18]. Currently, cerebrovascular disease is considered an important contributing factor to vascular cognitive impairment [19]. Furthermore, silent brain infarction provoked by small vessel disease, rather than large cortical cerebral infarction, is more important for the development of vascular cognitive impairment although various cardiovascular risk factors are related to the occurrence of both types of cerebral infarction [3, 4]. Brain tissue changes characterizing AD including amyloid *β* plaques and neurofibrillary pathology occur more often in patients with cerebrovascular disease than in healthy elderly people. As defined in the Nun study, a combination of Alzheimer type pathological changes and microvascular disease can worsen cognitive function in elderly persons [20]. Therefore, the development of small vessel disease has been shown to strongly influence the degree of cognitive function in patients with AD. In addition, several reports indicated that atherosclerosis in the carotid artery was associated with a prospective risk of cognitive impairment. Early intervention of carotid atherosclerosis may therefore be helpful in delaying or preventing the onset of cognitive impairment [21, 22].

In a recent Japanese study, high intake of *ω*3 PUFA was shown to have antiatherogenic effects reducing the nonfatal coronary events in statin-treated patients with coronary artery diseases [9]. In addition, *ω*3 PUFA and low dose statin combination therapy reduced the risk of recurrent stroke in Japanese patients with hyperlipidemia [10]. These two studies and their subanalyses demonstrate that PUFA balance is an important diagnostic parameter to evaluate the disease progression in arteriosclerotic diseases associated with coronary artery and cerebrovascular diseases.

Various trials in patients with cerebrovascular diseases have indicated that *ω*3 PUFAs intake brings beneficial effects in primary and secondary prevention [10, 23]. In a large cohort study of older adults, high consumption of marine *ω*3 PUFA reduced the prevalence of MRI-detected cerebral infarcts and WMH [8]. In addition, carotid intima-media thickness, reported to be a prospective risk parameter of cognitive impairment, was regressed in patients who took *ω*3 PUFA [24, 25]. Some investigations elucidated that patients with AD had low serum *ω*3 PUFA levels and this might have an etiological role in the pathogenesis of AD [26]. Some reports showed that administration of *ω*3 PUFA improved cognitive function in cases of mild cognitive impairment, although these studies did not include large patient cohorts [10–12]. Our investigation indicated that sufficient *ω*3 PUFA intake might be effective in preventing or postponing the future development of cognitive impairment in middle aged or presenile persons who do not have current cognitive decline but who do have certain atherosclerotic risk factors and serum *ω*3 PUFA abnormalities.

The therapeutic action of *ω*3 PUFA in vascular disease has been associated with antiinflammatory effects, the inhibition of platelet aggregation, the improvement of endothelial function, and plaque stabilization through the following actions [27–29]. PUFA is catalyzed through cyclooxygenases and lipoxygenases into several active eicosanoids including prostaglandins, thromboxanes, and leukotrienes and each interaction produces the physiological effects described above. EPA has 20 carbon chains in its structure and acts as an inhibitor of AA having the same 20 carbon chains [30]. From a biochemical position, EPA, having 20 carbon chains, is readily converted via both the cyclooxygenase and the 5-lipoxygenase pathways, but DHA having 22 carbon chains is not metabolized via either of these pathways. Ninomiya et al. reported that a low serum EPA/AA ratio is associated with a greater risk of cardiovascular disease in subjects with more high-sensitivity C reactive protein, but this association was not detected for the serum DHA/AA ratio [31]. In addition, in patients who underwent endarterectomy under administration of PUFA, a higher content of EPA was detected in the resected atherosclerotic plaques, but there was no difference in DHA content, compared to that in control subjects [32]. Therefore, EPA is not only a precursor of DHA, and there may be some differences in selectivity by phospholipase and metabolism in the arachidonic acid cascade between EPA and DHA, and these 2 *ω*3 PUFAs may show distinct therapeutic effects. In our study, only the reduction of EPA, and not DHA, was related to the progression of WMH, especially DSWMH.

The previous investigations revealed that brain had a unique PUFA composition with low level of EPA in contrast to high level of AA and DHA, and the lower level of EPA was maintained by several pathways including rapid metabolism by *β*-oxidation and lower recycling within brain phospholipids [33, 34]. Also, Freund Levi et al. evaluated the transfer of *ω*3 PUFAs (DHA-rich) to the brain by oral supplementation in patients with Alzheimer's disease (AD). So, their investigation revealed that EPA and DHA levels increased both in plasma and cerebrospinal fluid (CSF) and the increase of EPA in plasma was correlated with those in CSF levels, but there was no correlation between each DHA variation in plasma and CSF [35]. Furthermore, in this study there was an important observation that the more the DHA level increased in CSF the greater the change in AD biomarker (phosphorylated tau) in CSF. This study revealed that EPA transfer to the brain across the human blood brain barrier was much higher compared with DHA. These observations may also indicate a benefit of EPA supplement in patients with cognitive impairment.

Limitations of this study were as follows. The estimation of WMH was only semiquantitatively performed and this may potentially affect our ability to accurately estimate the true relationship between *ω*3 PUFA and WMH. The subjects with atherosclerotic factors, such as dyslipidemia, diabetes, or smoking, were underrepresented in the same population and these 3 risk factors were not related to the progression in WMH. This may be related to patient backgrounds, since half of the patients in the study had received various examinations for physical checkup. In addition, only FLAIR images were used for evaluating WMH, although we had data from T1 and T2 weighted images. Therefore, other ischemic lesions may be missed.

In conclusions, this study suggested that the progression of WMH in presenile patients was associated with a low serum EPA/AA ratio, as well as aging, female sex, hypertension, and the presence of ICA plaques, and accompanied minimal, but significant, deterioration in cognitive function. Sufficient *ω*3 PUFA intake may be useful in preventing cognitive impairment prior to clinical development.

## Supplementary Material

Supp Figure: The grading scale of white matter hyperintensity (WMH) released from the Japanese brain dock society (http://jbds.jp/guideline.html). This grading scale in deep and subcortical WMH (DSWMH) and periventricular WMH (PVH) was both consisted from 5 grades (DSWMH: grade 0 to 4 and PVH: grade 0 to IV) .
Supp Table: The raw data of 3 FUFAs and their ratios according to WMH levels in the two WMH types (DSWMH and PVH).

## Figures and Tables

**Table 1:** Factors associated with the progression of deep and subcortical white matter hyperintensity (DSWMH) grades using median values of PUFA.

Factors	DSWMH			
	Estimate	SE	Chi-square	*P* value
Age	−0.1217	0.0176	47.59	<0.0001
Female sex	−0.9703	0.2650	13.41	0.0003
Hypertension	−0.8629	0.2567	11.30	0.0008
Dyslipidemia	−0.1569	0.2447	0.41	0.5215
Diabetes	−0.3323	0.3353	0.98	0.3216
Smoking	−0.1907	0.2697	0.50	0.4795
ICA plaque	−0.9129	0.2608	12.25	0.0005
EPA/AA < 0.38	−1.4752	0.2900	25.88	<0.0001
DHA/AA < 0.84	−0.2798	0.2772	1.02	0.3128

**Table 2:** Factors associated with the progression of periventricular hyperintensity (PVH) grades using median values of PUFA.

Factors	PVH			
	Estimate	SE	Chi-square	*P* value
Age	−0.1534	0.0194	62.48	<0.0001
Female sex	−0.8844	0.2775	10.16	0.0014
Hypertension	−0.6569	0.2730	5.79	0.0161
Dyslipidemia	−0.1749	0.2612	0.45	0.5032
Diabetes	−0.5092	0.3536	2.07	0.1498
Smoking	−0.5132	0.2859	3.22	0.0726
ICA plaque	−0.5532	0.2748	4.05	0.0441
EPA/AA < 0.38	−1.1522	0.3072	14.06	0.0002
DHA/AA < 0.84	−0.4061	0.2994	1.84	0.1750

**Table 3:** Comparative data of Mini-Mental State Examination (MMSE) scores and background diseases between patients with advanced grade (grade ≥ 3 in DSWMH or grade ≥ III in PVH) and those with both no or mild grade in DSWMH and PVH.

Groups	A	B	C	*P* value
WMH	No or mild	Moderate	Advanced	
Grades	(0, I and 0, 1)	(II and 2)	(III, IV or 3, 4)	
Patients number	74	84	58	
Male/female	46/28	45/39	29/29	ns
Age	66.9 ± 5.3	67.5 ± 5.2	70.6 ± 6.3	*P* < 0.0008^*∗∗*^
				*P* < 0.005^*∗∗∗*^
MMSE scores	29.7 ± 1.0	29.3 ± 1.3	28.4 ± 2.1	*P* < 0.0001^*∗∗*^
				*P* < 0.002^*∗∗∗*^
EPA/AA	0.56 ± 0.20	0.36 ± 0.18	0.34 ± 0.24	*P* < 0.0001^*∗*,*∗∗*^
DHA/AA	1.0 ± 0.24	0.79 ± 0.22	0.78 ± 0.24	*P* < 0.0001^*∗*,*∗∗*^
Hypertension	43/31	60/24	48/10	
Dyslipidemia (+/−)	46/28	63/21	37/21	
Diabetes (+/−)	7/67	14/70	8/50	
Smoking (+/−)	22/52	30/54	23/35	
ICA plaque (+/−)	45/29	62/22	48/10	

^*∗*^Between groups A and B, ^*∗∗*^between groups A and C, and ^*∗∗∗*^between groups B and C.
