# Supplementary material for: The Association between Cerebral White Matter Lesions and Plasma Omega-3 to Omega-6 Polyunsaturated Fatty Acids Ratio to Cognitive Impairment Development
Source: Biomed Res Int. 2015 Oct 25;2015:153437. doi: 10.1155/2015/153437 (PMC4637015; doi:10.1155/2015/153437)
Supplement: Supplementary file 1 — Supp Figure: The grading scale of white matter hyperintensity (WMH) released from the Japanese brain dock society (http://jbds.jp/guideline.html). This grading scale in deep and subcortical WMH (DSWMH) and periventricular WMH (PVH) was both consisted from 5 grades (DSWMH: grade 0 to 4 and PVH: grade 0 to IV) . Supp Table: The raw data of 3 FUFAs and their ratios according to WMH levels in the two WMH types (DSWMH and PVH). [file 153437.f1.docx]

**Supplementary Materials**

- Supplement Figure


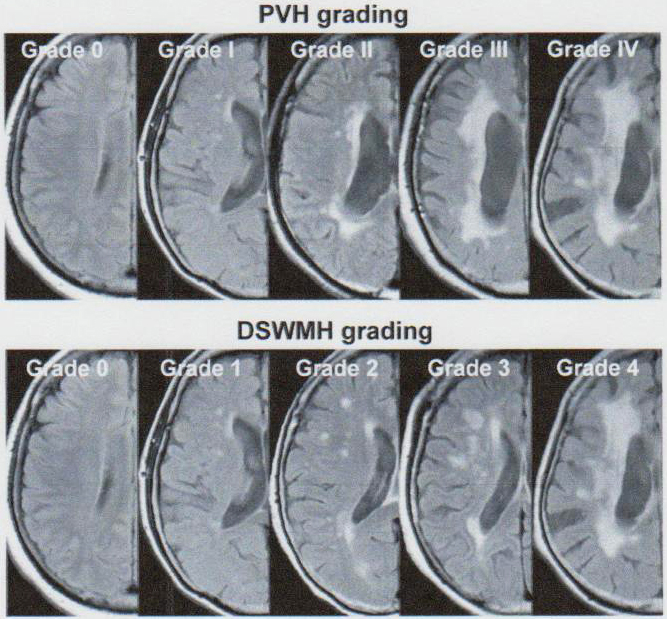


- Supplement Table

| WMH type DSWMH  Grades 0 I II III IV  Pts number 11 101 107 63 9 |
| --- |

EPA 89.64±65.22 95.06±40.16 73.81±39.16 62.43±29.38 62.52±18.05

DHA 159.49±61.70 179.46±54.36 159.34±49.98 151.89±52.65 139.44±29.99

AA 188.96±43.91 188.67±36.17 201.06±44.34 198.13±41.87 187.03±36.14

EPA/AA 0.48±0.30 0.52±0.23 0.38±0.20 0.33±0.23 0.35±0.15

DHA/AA 0.87±0.34 　0.96±0.29 0.80±0.27 0.78±0.24 0.76±0.15

| WMH type PVH  Grades 0 1 2 3 4  Pts number 30 161 84 8 8 |
| --- |

EPA 88.35±38.88 84.84±42.50 66.57±35.28 61.38±23.01 73.46±35.28

DHA 165.71±55.92 175.20±55.03 148.00±45.52 118.48±21.32 149.45±34.38

AA 197.92±41.18 192.91±41.23 200.14±41.34 188.65±36.90 186.86±39.10

EPA/AA 0.46±0.22 0.46±0.25 0.34±018 0.34±0.15 0.42±0.25

DHA/AA 0.84±0.26 0.93±0.30 0.74±0.23 0.65±0.17 0.82±0.22

|  |
| --- |
